# Road Rage: Prevalence Pattern and Web Based Survey Feasibility

**DOI:** 10.1155/2014/897493

**Published:** 2014-04-23

**Authors:** Shaily Mina, Rohit Verma, Yatan Pal Singh Balhara, Shiraz Ul-Hasan

**Affiliations:** ^1^Department of Psychiatry, Lady Hardinge Medical College & Smt. S. K. Hospital, New Delhi 110001, India; ^2^Department of Psychiatry, All India Institute of Medical Sciences, New Delhi 110029, India

## Abstract

*Introduction.* Incidents of road rage are on a rise in India, but the literature is lacking in the aspect. There is an increasing realization of possibility of effective web based interventions to deliver public health related messages. * Objective.* The aim was to quantitatively evaluate risk factors among motor vehicle drivers using an internet based survey. * Methods*. Facebook users were evaluated using Life Orientation Test-Revised (LOT-R) and Driving Anger Scale (DAS). * Results.* An adequate response rate of 65.9% and satisfactory reliability with sizable correlation were obtained for both scales. Age was found to be positively correlated to LOT-R scores (*r* = 0.21; *P* = 0.02) and negatively correlated to DAS scores (*r* = −0.19; *P* = 0.03). Years of education were correlated to LOT-R scores (*r* = 0.26; *P* = 0.005) but not DAS scores (*r* = −0.14; *P* = 0.11). LOT-R scores did not correlate to DAS scores. * Conclusion.* There is high prevalence of anger amongst drivers in India particularly among younger males. A short web survey formatted in easy to use question language can result in a feasible conduction of an online survey.

## 1. Introduction

Aggressive driving has been defined as that which endangers or is likely to endanger people or property by the National Highway Traffic and Safety Administration [1]. It includes tailgating, abrupt lane changes, and speeding, alone or in combination. On the other hand, road rage is an event on roadways where an angry or impatient vehicle driver or passenger intentionally attempts, threatens to injure, injures, or kills another vehicle driver, passenger, or pedestrian in response to a traffic dispute, altercation, or grievance [2]. It includes verbal nuisance, threats, obscene gesturing, flashing headlights, or high-beams, horn honking, malicious braking, blocking other vehicles, threatening with weapons, firing gun shots, hitting vehicles with objects, chasing a vehicle, and trying to run a vehicle off the road and is considered a criminal offence [3].

These two terms have also been considered to be a continuum. Shinar [4] defines four levels of aggression; first is nonthreatening gestures such as yelling; second is aggressive driving such as using obscene language or excessive horn honking/light use; third is mild road rage such as threatening behaviour; and last is extreme road rage which includes direct confrontation. Literature reports two broad categories of aggressive behaviour: hostile and instrumental. Hostile (“reactive, impulsive, or affective”) behaviour involves behaviour intended to make the aggressor feel contented without thinking of the consequences whereas instrumental (“proactive, premeditated, or predative”) behaviour aims to overcome the frustrating situation or event [5, 6]. Research is still undergoing to develop more precise definition of these terms.

Road rage increases crime rates in form of arguments and assaults ending up in injury and even death. Risky driving is one of the most common causes of road traffic accidents [7]. The prevalence of road traffic accidents (RTA) has been increasing drastically every year. It is estimated that, by 2020, road traffic disability-adjusted life years lost will move from being the 9th to the 3rd leading cause in the world and 2nd leading cause in developing countries [8]. According to WHO, RTAs are the sixth leading cause for hospitalization, disabilities, death, and economic losses in India [9].

Factors which can lead to road rage can be traffic congestion, weather condition, noise, time constraint, underlying emotions (anger, frustration, and irritation), individual characteristics (age, gender, socioeconomic status, and culture), and personality of the person responsible for road rage. Social learning theorists suggest that it is a response learned through observation or imitation of significant social figures in their life, media, or on road experiences [10].

Despite the increased prevalence of road rage, there have been very few studies being done so far limited to developed country to assess the risk factors profile of people responsible for the same. Such study is necessary especially in developing countries like India which is having favourable factors for road rage like overcrowding, nonstringent traffic rules.

Usage of internet is rapidly increasing day by day worldwide [11], and much of this activity has been focused on health. The public health impact of an intervention is defined by the program's efficacy multiplied by its reach [12]. With the growing internet use, web based programmes (WBP) have a potential to reach a large section of the population at lower costs than face-to-face interventions [13]. Moreover, by using the internet, participants can access large amounts of information, and they can choose the time when they would like to interact and receive information [14]. However, few studies have pointed out that the actual use of WBP may be limited [15, 16] and that leaving a WBP prematurely is common [17, 18]. The qualitative value of WBP may also be hindered by the fact that having WBP at health care setting may make participant compelled to fill out forms rather than when using the same service at home. Prior to developing an effective intervention to manage the targeted health condition, an assessment of its prevalence and feasibility of action through internet needs to be done.

Web 2.0 has allowed the creation of an online environment where users share knowledge and information and build friendship or interest communities [19]. This shift to a user-led environment is reflected in the list of global top 10 visited sites on the web wherein Facebook is topmost among the social networking websites making it an important means to assess and deliver public health related messages [20].

Current study is the first web based study quantitatively evaluating risk factors through validated scales among motor vehicle drivers in India. Such study can be used to control or modify the risk behaviours and strengthen the existing policies on road safety measures, education, and health in the country. It might help broaden the means to reduce or circumvent consequences of road traffic accidents related to road. It can also be used to assess the feasibility of internet as means to assess public health.

## 2. Material and Method

This pilot cross-sectional web based study was conducted from May 2013 to August 2013.

### 2.1. Participants and Procedure

The cases in the study population were selected from Facebook. A random selection of Delhi based Facebook user was made and the selected individual was approached over web for seeking permission to contact his friend list for participation in the study. After obtaining a written informed consent from him, all 18–50-year-old males in his friend list residing in Delhi were sent mail invitations for filling a survey proforma for assessing rage during driving.

The mail sent to the subjects consisted of detailed information about the study, reference of the primary contact, and a link to the study proforma generated by the authors for assessment. All subjects who had not responded to the first email were sent a second and final mail offering to participate in the study or provide the reason for not participating.

The web proforma consisted of three pages of information gathering procedure. Prior to filling the assessment proforma, subjects were to provide written web based consent for participation in the study. Only subjects providing the written consent could further proceed and fill the study proforma. The first page consisted of semistructured format for collecting sociodemographic data. The subsequent pages consisted of the assessment instruments. The procedure and instruments were kept as short as possible for better participation in the study.

### 2.2. Assessment Instruments

#### 2.2.1. Semistructured Proforma

This was used to collect data regarding sociodemography and parameters related to driving such as type of vehicle driven or years of driving.

#### 2.2.2. Life Orientation Test-Revised (LOT-R)

LOT is designed to express generalized expectations of positive life events and measures dispositional optimism [21]. It is a self-report questionnaire assessing an individual's tendency to expect positive compared to negative outcomes. LOT has 12 items consisting of 8 items (four fillers) and reflects a single bipolar dimension in which higher scores indicate greater optimism or less pessimism. Conversely, the LOT optimism and pessimism subscale scores reflect separate unipolar dimensions. The revised version (LOT-R) has 10 items (six-item measure with four additional filler items) with 3 items being positively phrased (e.g., “I'm always optimistic about my future”) and 3 items being negatively phrased (e.g., “I hardly ever expect things to go my way”) [22]. Participants rated each item by indicating the extent of their agreement along a 5-point Likert scale, ranging from “strongly agree” to “strongly disagree.” Items are summed to produce a total score (from 0 to 24 points) as well as separate optimism and pessimism subscale scores. Overall, the higher the score is, the higher the level of optimism is reported. Research has demonstrated test-retest reliability of 0.68, 0.60, 0.56, and 0.79, at four, twelve, twenty-four, and twenty-eight months, respectively [22].

#### 2.2.3. Driving Anger Scale (DAS)

The DAS measures driver anger dimension with potential value for research on accident prevention and health psychology [23]. The DAS correlates positively with intensity of anger and frequency of anger, aggression, and risky behavior while driving, aggressive expression of driving anger, and general trait anger [24]. Items present common driving scenarios that have been found to elicit varying degrees of anger. For the purpose of the present study, participants were asked to imagine that each scenario had just happened during their commute and rate the level of anger they would have experienced using a 1–5 Likert scale (1 = “not at all” and 5 = “very much”). The original DAS was a 33-item scale (alpha reliability = 0.90) with six reliable subscales involving hostile gestures (DAS-HG), illegal driving (DAS-ID), police presence (DAS-PP), slow driving (DAS-SD), discourtesy (DAS-D), and traffic obstructions (DAS-TO). A 14-item short form was found to demonstrate good reliability (alpha = 0.80–0.93) and to correlate highly with the long form of the DAS (*r* = 0.95) [23]. A recent 10-week test-retest reliability of short form was found to be 0.84 [24]. The current study utilized the short form of DAS. Scoring consisted of summing the responses to the scores on individual subscales and a total summated response of 14 items, with higher scores representing greater driver anger.

### 2.3. Analysis

Data was imputed and analysed using SPSS ver. 17.0. ANOVA was done to determine in-between group differences on basis of type of vehicle driven, age, and bipolar optimism-negativism trait. Phi and Cramer's test were done to evaluate group differences based on education and years of driving. Internal consistency of DAS was assessed by Cronbach's alpha coefficient and values of 0.70 or greater were considered satisfactory. Pearson's correlation was used to calculate the correlation between different questionnaires and other variables like sociodemographic parameters, years of driving, driving professionalism, and type of vehicle driven. A hierarchical entry multiple regression was conducted for predicting DAS scores.

## 3. Results

Four hundred and thirty-seven individuals were found to be friends of the primary Facebook contact, out of whom inclusion criteria were met by 176 individuals to whom the mail invitations were then sent. A total of 106 subjects returned the questionnaire. On resending the mail, 10 more subjects participated in the study, but the rest 60 individuals, who had not responded to the first mail, provided no reply on the resent mail. Finally, data of 116 subjects was analysed with SPSS 17.0.

The web proforma was designed in such a way that in order to fill the survey proforma subject needed to sign the web consent form. Thus, all subjects had provided the consent for participation in the study.

All the subjects were male with a mean age of 27.09 ± 6.73 years (range 16–55). About two-thirds of the subjects had completed graduation or postgraduation (*n* = 87) and the majority were driving for more than 4 years (Table 1).

All participating subjects responded to all the questions of web proforma (Table 2). The mean score of LOT-R was 14.11 ± 2.93 (range 8–21) and total DAS score was 44.09 ± 9.73 (range 23–65).

Table 3 contains all bivariate correlations, means, and alpha reliabilities for DAS scale and subscales. Overall, all measures demonstrated moderate to high reliability (*α* = 0.72–0.76). Significant relationships (*P* < 0.01) were found between the individual DAS subscales and total scores except for DAS-TO and DAS-ID (*r* = 0.15, *P* = 0.09). For individual items of DAS, Cronbach's alpha ranged from 0.75 to 0.79 demonstrating moderate to high reliability close to the original values reported by Deffenbacher.

In the present sample, internal consistency reliability was moderate to high for the LOT-R total scale and each subscale according to Cronbach's coefficient alphas: total (*α* = 0.65); optimism (*α* = 0.84); and pessimism (*α* = 0.68). The Pearson correlation coefficient between the two subscales was sizable (*r* = −0.74, *P* < 0.01).

An optimistic orientation was noted in 44% subjects and negativism in 56% subjects, after summating total LOT-R score of 15 or more as optimism. There was no difference on any assessment parameter on basis of LOT-R scores nor the scores correlated to DAS scores.

Age was found to be positively correlated to LOT-R scores (*r* = 0.21, *P* = 0.02) and negatively correlated to DAS scores (*r* = −0.19, *P* = 0.03). Years of education were correlated to LOT-R scores (*r* = 0.26, *P* = 0.005) but not DAS scores (*r* = −0.14, *P* = 0.11).

Drivers of two wheelers were of significantly lesser age as compared to that of four wheelers (24.98 ± 6.10 and 29.50 ± 6.66, resp., *P* < 0.0001). Though total DAS scores were higher (46.79 ± 8.85 and 41.00 ± 9.86, resp., *P* = 0.001), there was no difference on subscale scores of hostile gestures and illegal driving (Table 4). There were no differences in the two types of driver groups on LOT-R scores or any other parameters.

No differences were found on basis of driving as a profession or years of driving.

In order to examine the influences of driver optimistic orientation on road rage, separate hierarchical entry multiple regressions were conducted for DAS scores. The procedure was to enter demographics (age and education) in the first step, years of driving in the second step, driving as profession in third step, and DAS scores in the fourth step and to add all cross product interactions stepwise in the fifth step. Except for the first step (beta = 0.067,* t* = 1.66, Δ*R* ^2^ = 0.045, Δ*F* = 5.41, *P* = 0.02), no prediction for DAS score was found.

## 4. Discussion

### 4.1. Feasibility of Online Survey

The current study is a pilot web based survey evaluating risk factors of road rage in India. Although participation in online surveys is supposed to be easy for frequent computer users [25], one major concern is the online surveys' low response rates. On average, online survey response rates are 11% below mail and phone surveys with reports of rates as low as 2% [26]. Paper based surveys are reported to be having more response rates than online ones. In the research by Watt et al. [27], the overall response rate for online surveys was 32.6%, while for paper surveys it was 33.3%. Multiple factors are involved to explain these low rates such as reminder mails, incentives, duration of availability, length of survey, and anonymity [28]. It is suggested that response rates of 60% or more are both desirable and achievable for online surveys [29]. We achieved a response rate of 65.9% in the study which can be labelled as an adequate response. There was satisfactory reliability and sizable correlation obtained for both the scales (DAS: *α* = 0.73; LOT-R: *α* = 0.65). Thus we could attribute our study methodology to signify a feasible opportunity to conduct an online survey utilizing a social networking website.

### 4.2. Factors Affecting Road Rage

On comparison of the DAS subscales, we found that police presence was the least anger provoking situation as observed in other Indian and western studies [30, 31]. Traffic obstruction scenario caused highest level of anger whereas discourtesy was observed to be causing higher anger in other similar studies on driver rage [30, 31]. Reason for this disparity could be that the study conducted in India was from a less crowded city compared to a metro city like Delhi wherein cases for our study were taken. Overcrowding could have led to finding of more traffic obstruction in our study. Developed countries have more stringent traffic rules and less overcrowding which could have led to discourtesy being more than traffic obstruction as a cause for anger in drivers.

On assessment of DAS subscale scoring with other countries like US, UK, and Australia, anger levels were more in India in all the subscales. Researchers have attempted to explain the reason for such behaviour by frustration-aggression theory which implies that congestion or delays are “goal blocking,” interfering with driving progress [4]. Drivers experience an increase in frustration that in turn lowers the driver's aggression threshold increasing the likelihood of road aggression. Tolerance to anger provoking situation is decreasing day by day especially in developing countries like India due to callous attitude of both the drivers and the pedestrians to follow traffic rules. Overcrowding of both vehicles and people leads to more of unauthorized parking and traffic congestion. Also the long working hours and extreme hot weather conditions in India may be reducing threshold for anger among drivers.

Deffenbacher [32] found anger to be positively correlated with coping strategies adapted by an individual and was less likely to express their anger through prosocial, adaptive/constructive means when exposed to anger provoking situation. In our study, though more than half of the subjects had negativistic view when dealing with the situation which would increase anger, no significant correlation of anger was found with expectation.

While taking into account the age, we found more anger levels in younger drivers. This finding is supported by previous studies mentioning young male drivers to be approximately three times more risky than mature adults increasing their proneness to untoward consequences [33]. Golias and Karlaftis [34] observed that when age is considered, drivers seem to be more law abiding and less risk taking as they grow older, therefore decreasing chances of anger levels and road rage. Some authors report reckless, faster, and dangerous driving to be more frequent in young drivers increasing the chances of road rage [35, 36]. Age has been quoted as the most important factor in aggressive driving incidents [37], with majority of aggressive drivers being men between the ages of 18 and 26 [38]. Maximum aggressive responses were shown by the respondents of 19- to 25-year age group in another Indian study [39]. Sensation seeking and aggressiveness have been significantly correlated with younger drivers when assessing the personality [37]. Other reasons of more anger levels in young drivers could be less experience to deal with such situation, influence of substance taking, peer influence, and reduced attention span [40].

Current study found no differences on basis of driving as a profession or years of driving. This is in accordance with the research by Chomeya [41] showing no significance on comparison of learning achievement, driving confidence, and driving experience. The reason could be that samples were not much different in their ages and development of maturity in diverse situation. Contrasting findings were seen in another study showing driver having new driving experiences to be having maximum chances for involving in road rage incidences as compared to the other professional categories [42].

Current study found no correlation with anger and the trait characteristic of the driver (optimism or pessimism), probably suggesting that external factors influence anger level more than internal factors like behaviour of other drivers, dense traffic, and being interrupted or cut off by another driver. This finding is supported by showing influence of environmental factors to be more significant in modulating anger level [43]. Hennessy and Wiesenthal [44] reviewed driver trait dispositions for stress and their self-reported stress levels during periods of high and low congestion on the roads. Regardless of an individual's trait disposition for stress, all drivers reported higher stress in the high congestion condition. This finding further supports that impact of external factors is higher. However, contrary views have also been observed finding negative attributions or negative evaluations/thoughts affecting aggressive reactions to other drivers' provocative road behaviour [45].

Age was positively correlated with constructive view about any situation with young drivers being more pessimistic than older drivers. Previous literature also reports similar findings showing young male drivers having higher overall self-reported life stress scores [46]. Negative life events result in more aggressive behaviour in youth compared to older adults [47].

On assessment of optimistic orientation, more than half of the participants had negativistic view regarding the future. Such individuals have higher propensity to have amplified stress levels. Similar inference was made by McMurray [48] mentioning propensity to be engaged in risk taking behaviour such as reckless driving; ventilating anger towards others is more in people with higher trait for developing stress and using a vehicle as an outlet for their stress.

The level of education attained has been found to have a negative relationship with individual tolerance for aggression [49]. In the current study such inference cannot be made due to heterogeneous sample with majority of participants being educated.

There are few measures still under research for reducing the incidence of road rage, like in Europe where compulsory classes are arranged for drivers to warn of the consequences of aggressive and inconsiderate driving. Traffic psychologist has suggested modifying the car design to prevent road rage such as cars that stop drivers from constantly flashing their headlights or sounding the horn. Some cars have even been designed to include a sensor located in the front grill that detects the distance between the car and the car in front and modifies speed accordingly [40]. But such provisions are not feasible in developing countries like India. The following measures can be undertaken: education programmes for traffic rules before providing licence, more stringent rules for producing licence and following traffic rules, invocation of penalty and jail terms for breaking rules, and better technology to detect red light jumpers. Sessions should be arranged for drivers with focus on emotional management by way of listening to songs, controlling anger, distracting oneself, and so forth.

Future studies should target a larger population for online assessment comparing multiple cities and drivers in view of developing a web based programme to curtail anger in drivers and suggesting policy makers to make appropriate reforms in traffic regulations for better road situations.

## 5. Conclusion

There is high prevalence of anger amongst drivers in India. Internet can be used as a means to evaluate anger related to driving in India utilizing social networking sites. A short web survey formatted in easy to use question language with ability to view forms in different browsers, personalized reminders, and support of a primary contact/gatekeeper can result in a feasible conduction of an online survey.

## Figures and Tables

**Table 1:** Sociodemographic variables.

Parameter	Frequency	%
Education		
Illiterate	2	1.7
Primary	—	—
Secondary	6	5.2
Higher secondary	21	18.1
Graduate	48	41.4
Postgraduate	39	33.6
Number of driving years		
Less than 6 months	6	5.2
6 months–1 year	8	6.9
2-3 years	16	13.8
4-5 years	32	27.6
6–10 years	28	24.1
More than 10 years	26	22.4
Type of vehicle driven		
Two-wheeler	62	54.3
Four-wheeler	54	45.7
Professional driver		
No	107	92.2
Yes	9	7.8

**Table 2:** Responses to DAS situations by study population.

Number	DAS situations	Perceived anger^#^				
		1	2	3	4	5
1	Someone is weaving in and out of traffic	13.8	23.3	27.6	15.5	19.8
2	A slow vehicle on a mountain road will not pull over and let people by	12.1	35.3	18.1	19.0	15.5
3	Someone backs right out in front of you without looking	6.9	15.5	23.3	29.3	25.0
4	Someone runs a red light or stop sign	11.2	28.4	22.4	21.6	16.4
5	You pass a radar speed trap	23.3	30.2	19.0	11.2	16.4
6	Someone speeds up when you try to pass him/her	23.3	25.0	21.6	15.5	14.7
7	Someone is slow in parking and is holding up traffic	12.9	27.6	19.0	18.1	22.4
8	You are stuck in a traffic jam	6.0	20.7	17.2	13.8	42.2
9	Someone makes an obscene gesture toward you about your driving	7.8	29.3	12.1	20.7	30.2
10	Someone honks at you about your driving	9.5	24.1	12.1	23.3	31.0
11	A bicyclist is riding in the middle of the lane and is slowing traffic	11.2	27.6	19.0	22.4	19.8
12	A police officer pulls you over	17.2	20.7	22.4	18.1	21.6
13	A truck kicks up sand or gravel on the car you are driving	9.5	20.7	16.4	19.0	34.5
14	You are driving behind a large truck and you cannot see around it	15.5	22.4	27.6	14.7	19.8

Driving Anger Scale (DAS); ^#^amount of anger perceived is quantified as none at all = 1; a little = 2; some = 3; much = 4; and very much = 5. All the values are in percentages.

**Table 3:** Intercorrelations, means, standard deviations, and reliabilities for Driving Anger Scale.

	DAS-HG	DAS-ID	DAS-PP	DAS-SD	DAS-D	DAS-TO	DAS Total
DAS-HG	—	0.28**	0.49**	0.38**	0.43**	0.48**	0.76**
DAS-ID	—	—	0.25**	0.24**	0.47**	0.15	0.55**
DAS-PP	—	—	—	0.34**	0.45**	0.33**	0.68**
DAS-SD	—	—	—	—	0.41**	0.32**	0.62**
DAS-D	—	—	—	—	—	0.35**	0.76**
DAS-TO	—	—	—	—	—	—	0.69**
DAS Total	—	—	—	—	—	—	—
Mean	6.78	6.08	5.73	6.00	9.35	10.15	44.09
SD	2.50	2.01	2.01	1.85	2.68	3.02	9.73
Alpha	0.72	0.75	0.74	0.75	0.72	0.73	0.76

Driving Anger Scale (DAS); hostile gestures (DAS-HG); illegal driving (DAS-ID); police presence (DAS-PP); slow driving (DAS-SD); discourtesy (DAS-D); traffic obstructions (DAS-TO); standard deviation (SD); **P < 0.01.

**Table 4:** Group comparison on basis of driving vehicle [two-wheeler (*n* = 62) and four-wheeler (*n* = 54)].

Parameter	Two-wheeler Mean ± SD	Four-wheeler Mean ± SD	*P* value	Confidence interval	
				Lower	Higher
Age	24.98 ± 6.10	29.50 ± 6.66	<0.0001**	−6.86	−2.16
LOT-R	13.69 ± 2.75	14.59 ± 3.08	0.100	−1.97	0.17
DAS total	46.79 ± 8.85	41.00 ± 9.86	0.001**	2.34	9.23
DAS-HG	7.19 ± 2.31	6.31 ± 2.64	0.059	−0.03	1.79
DAS-ID	6.26 ± 1.92	5.87 ± 2.10	0.302	−0.35	1.12
DAS-PP	6.15 ± 1.92	5.26 ± 2.03	0.017*	0.15	1.61
DAS-SD	6.37 ± 1.86	5.57 ± 1.76	0.020*	0.12	1.46
DAS-D	9.95 ± 2.69	8.67 ± 2.53	0.010*	0.31	2.25
DAS-TO	10.87 ± 2.72	9.31 ± 3.16	0.005**	0.47	2.65

Driving Anger Scale (DAS); hostile gestures (DAS-HG); illegal driving (DAS-ID); police presence (DAS-PP); slow driving (DAS-SD); discourtesy (DAS-D); traffic obstructions (DAS-TO); life optimism test-revised (LOT-R); standard deviation (SD); *P < 0.05, **P < 0.01.

## References

[1] Martinez R (July 1997). *Testimony to House Transportation and Infrastructure Committee*.

[2] Mizell L (1997). *Aggressive Driving in Aggressive Driving: Three Studies*.

[3] Sarkar S, Martineau A, Emami M, Khatib M, Wallace K Spatial and temporal analyses of the variations in aggressive driving and road rage behaviours observed and reported on San Diego freeways.

[4] Shinar D (1998). Aggressive driving: the contribution of the drivers and the situation. *Transportation Research F: Traffic Psychology and Behaviour*.

[5] Barratt ES, Slaughter L (1998). Defining, measuring, and predicting impulsive aggression: a heuristic model. *Behavioral Sciences & the Law*.

[6] Houston RJ, Stanford MS, Villemarette-Pittman NR, Conklin SM, Helfritz LE (2003). Neurobiological correlates and clinical implications of aggressive subtypes. *Journal of Forensic Neuropsychology*.

[7] James L, Nahl D (2000). *Road Rage and Aggressive Driving: Steering Clear of Highway Warfare*.

[8] Murray CJL, Lopez AD (1997). Alternative projections of mortality and disability by cause 1990–2020: Global Burden of Disease Study. *The Lancet*.

[9] Ministry of Health and Family Welfare (2004). *Integrated Disease Surveillance Project: Project Implementation Plan 2004–09*.

[10] Grey EM, Triggs TJ, Haworth NL Driver Aggression: the role of personality, social characteristics, risk and motivation.

[11] Internet World Stats World Internet Users and Population Statistics 2012. http://www.internetworldstats.com/stats.htm.

[12] de Vries H, Brug J (1999). Computer-tailored interventions motivating people to adopt health promoting behaviours: introduction to a new approach. *Patient Education and Counseling*.

[13] Marcus BH, Nigg CR, Riebe D, Forsyth LH (2000). Interactive communication strategies: implications for population-based physical-activity promotion. *American Journal of Preventive Medicine*.

[14] Napolitano MA, Marcus BH (2002). Targeting and tailoring physical activity information using print and information technologies. *Exercise and Sport Sciences Reviews*.

[15] Leslie E, Marshall AL, Owen N, Bauman A (2005). Engagement and retention of participants in a physical activity website. *Preventive Medicine*.

[16] Glasgow RE (2007). eHealth evaluation and dissemination research. *American Journal of Preventive Medicine*.

[17] Evers KE, Cummins CO, Prochaska JO, Prochaska JM (2005). Online health behavior and disease management programs: are we ready for them? Are they ready for us?. *Journal of Medical Internet Research*.

[18] Brouwer W, Oenema A, Crutzen R, de Nooijer J, de Vries NK, Brug J (2009). What makes people decide to visit and use an internet-delivered behavior-change intervention? A qualitative study among adults. *Health Education*.

[19] O’Reilly T What is Web 2.0. http://oreilly.com/web2/archive/what-is-web-20.html.

[20] Alexa Internet About the Alexa Traffic Rankings. http://www.alexa.com/topsites.

[21] Scheier MF, Carver CS (1985). Optimism, coping, and health: assessment and implications of generalized outcome expectancies. *Health Psychology*.

[22] Scheier MF, Carver CS, Bridges MW (1994). Distinguishing optimism from neuroticism (and trait anxiety, self-mastery, and self-esteem): a reevaluation of the Life Orientation Test. *Journal of Personality and Social Psychology*.

[23] Deffenbacher JL, Oetting ER, Lynch RS (1994). Development of a driving anger scale. *Psychological Reports*.

[24] Deffenbacher JL, Huff ME, Lynch RS, Oetting ER, Salvatore NF (2000). Characteristics and treatment of high-anger drivers. *Journal of Counseling Psychology*.

[25] Israel GD (2011). Strategies for obtaining survey responses from extension clients: exploring the role of e-mail requests. *Journal of Extension*.

[26] Petchenik J, Watermolen DJ (2011). A cautionary note on using the internet to survey recent hunter education graduates. *Human Dimensions of Wildlife*.

[27] Watt S, Simpson C, Mckillop C, Nunn V (2002). Electronic course surveys: does automating feedback and reporting give better results?. *Assessment & Evaluation in Higher Education*.

[28] Nulty DD (2008). The adequacy of response rates to online and paper surveys: what can be done?. *Assessment & Evaluation in Higher Education*.

[29] Richardson JTE (2005). Instruments for obtaining student feedback: a review of the literature. *Assessment & Evaluation in Higher Education*.

[30] Lajunen T, Parker D, Stradling SG (1998). Dimensions of driver anger, aggressive and highway code violations and their mediation by safety orientation in UK drivers. *Transportation Research F: Traffic Psychology and Behaviour*.

[31] Dixit S, Deepa R, Bhagwat AK (2011). Road rage menace: a cross-sectional study to assess driver anger level in public motor vehicle drivers in a city in central India. *Online Journal of Health and Allied Sciences*.

[32] Deffenbacher JL (2003). Angry college student drivers: characteristics and a test of state-trait theory. *Psicologia Conductual*.

[33] Eksler V, Lassarre S, Thomas I (2008). Regional analysis of road mortality in Europe. *Public Health*.

[34] Golias I, Karlaftis MG (2001). An international comparative study of self-reported driver behavior. *Transportation Research F: Traffic Psychology and Behaviour*.

[35] Arnett JJ, Offer D, Fine MA (1997). Reckless driving in adolescence: “state” and “trait” factors. *Accident Analysis & Prevention*.

[36] Mitchell-Taverner P, Zipparo L, Goldsworthy J (2003). Survey on speeding and enforcement. *Report CR*.

[37] Arnett JJ (1994). Sensation seeking: a new conceptualization and a new scale. *Personality and Individual Differences*.

[38] American Automobile Association (1997). *‘Road Rage” on the Rise*.

[39] Chakrabarty N, Riku R (2013). Aggressive driving case studies and mitigations in India. *International Journal of Scientific and Research Publications*.

[40] Asbridge M, Smart RG, Mann RE (2006). Can we prevent road rage?. *Trauma, Violence, & Abuse*.

[41] Chomeya R (2010). Aggressive driving behaviour: undergraduate students study. *Journal of Social Sciences*.

[42] Gangopadhyay S, Ramalingaiah AN, Chakrabarty N (2009). *Understanding Road Rage: Implementation Plan for Promising Mitigating Measures*.

[43] Smith-Jackson TL, Wogalter MS, Shaver EF Road rage: user-reported antecedents and potential solutions.

[44] Hennessy DA, Wiesenthal DL (1997). The relationship between traffic congestion, driver stress and direct versus indirect coping behaviours. *Ergonomics*.

[45] Yagil D (2001). Interpersonal antecedents of drivers' aggression. *Transportation Research F: Traffic Psychology and Behaviour*.

[46] Simon F, Corbett C (1996). Road traffic offending, stress, age, and accident history among male and female drivers. *Ergonomics*.

[47] Aseltine RH, Gore S, Gordon J (2000). Life stress, anger and anxiety, and delinquency: an empirical test of general strain theory. *Journal of Health and Social Behavior*.

[48] McMurray L (1970). Emotional stress and driving performance: the effects of divorce. *Behavioural Research in Highway Safety*.

[49] Harris MB, Knight-Bohnhoff K (1996). Gender and aggression I: perceptions of aggression. *Sex Roles*.

